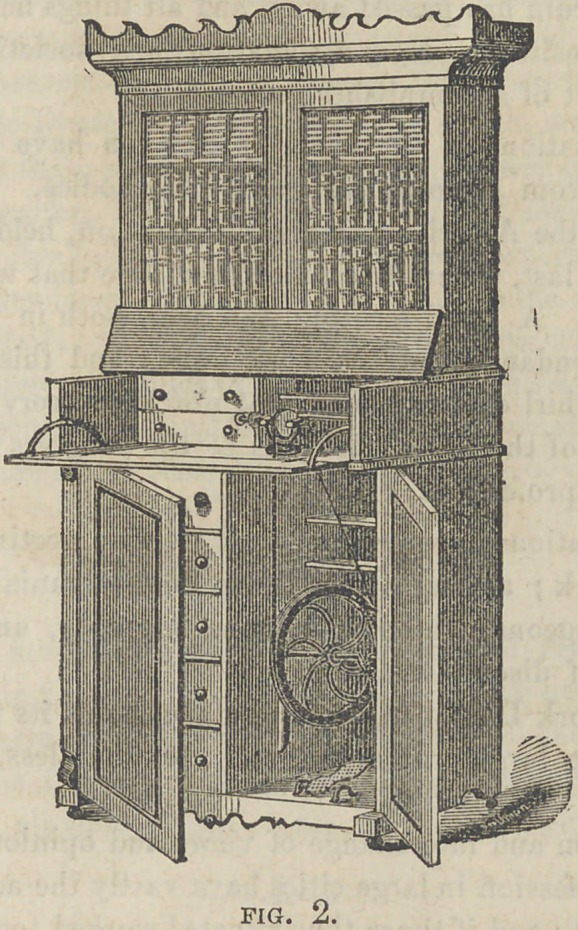# Editorial

**Published:** 1861-12

**Authors:** 


### Editorial.

### AMERICAN JOURNAL OF DENTAL SCIENCE.

The inquiry, where is the American Journal of Dental Science? produces a feeling of sadness—is it no more? only among the things that were? We have looked and looked for its coming, but it came not. It lived but a few days after him by whom it was produced, and by whom it lived. It was identified with Dr. Harris, and lived while he lived, and died when he died. Could there be none found to breathe into it the same spirit it possessed before? The seventeen volumes of the American Journal of Dental Science constitute a great and enduring monument of the editor and author, who, amid other cares, and toils and labors, carried on such a work. Shall it be said that the American Journal accomplished its destiny, or that it accomplished all the work there was to do? This at least could not have been affirmed had the author lived. We know not who would undertake such a work. We can hardly say that we wish to see the American Journal again; sorry indeed would we be to see that name associated with a less efficient and powerful mind than that which hitherto conducted it. It stands now a representation and embodiment of the art and science of the dental profession during the years of its publication, and though it may never again be published, its influence, like that of its noble author, will run down through all time.

T.

### TEETH.

We have recently been using teeth from the manufactory of D. H. Porter, of Bridgeport, Conn. These teeth we have never seen excelled; we have used more of his continuous-gum teeth than any other kind. This is the most trying kind of work upon teeth; it will blister or break those that are defective.

These teeth have passed through several heatings without changing, or sustaining the least injury; and we have seen none that are better formed or colored, or that look more life-like. The gum-teeth and those in sections for rubber work are very fine. These teeth require only to be introduced to the profession to come into general use. Hitherto we have had no facilities for obtaining these teeth except direct from the manufacturer. We hope, however, that arrangements will be soon made to supply our market. T.

### CLOSE OF THE VOLUME

With this number closes the fifteenth volume of the Register. It started out under favorable auspices, but it, like everything else of the kind, has been overtaken by an evil time. Contributors are less in number, and less inclined to write than formerly; subscribers are less numerous, and either have less money, or hold more tightly to it, than before. We hope, however, that notwithstanding all these discouraging circumstances, the Register has accomplished some good; we do not claim that it is the embodiment of dental science. We are willing the profession should set their own estimate on it. For the future, we can only say the Register will pursue the even tenor of its way, flourishing and rejoicing, in proportion to the sustenance it receives from the pens and pockets of our professional brethren. T.

### PERSONAL.

Perhaps we should have stated before this some facts and circumstances concerning our friend J. T. Toland. The profession generally, or in the West at least, are doubtless aware that he is not now, in person, looking after their interests in the way of dental supplies. His patriotism so expanded that a Dental Depot couldn't hold him; he therefore placed the dental business in competent hands, and off to the war, where wearing the garb and wielding the authority of Lieutenant Colonel, he fights for his country, striking hard blows, with a heavy hand, upon rebels wherever found. Than *he* none are braver.

Every one who knows John knows he will fight. The Dental Depot is under the best management, and as ever, supplies the profession, in its every demand, with the best material obtainable, and will continue to do so. T.

### CASE FOR OFFICE LATHE.

The accompanying cuts represent a dental lathe case, devised and planned by our friend Dr. H. R. Smith, of this city. We have thought an illustration and description of it might be of interest. Fig. 1 represents the case entirely closed, and shows it as a beauti

FIG. 1.

ful piece of furniture. Fig. 2 represents the case as thrown open, so far as the lathe departments are concerned, and from it we will say a few things. It is made in three sections; the lower is 3 feet 8 inches high, and 3 feet 6 inches wide, and 20 inches deep. It is divided in the center by a partition, one side of which is occupied by a tier of drawers, five in number. These are for storing dentifrices, napkins, cotton, wax, etc., etc.

On the other side of the partition is the wheel, treadle, etc. of the lathe. The front of this section is closed by two doors; upon this is placed the second section, the front of which is 9 inches high, and drops forward, in the same manner as the old style secretary; this, together with the inner part, constitutes the platform, upon which the lathe is placed.

There is back of the lathe a shelf and drawers, for small tools, teeth, etc. The top, to the width of 10 inches, is attached with hinges, and raises up at the same time that the front falls forward. One of St. John's adjustable lathes is used. This may be closed up by bringing down the top, and raising up the front, which are secured together by a lock.

The third section consists of a book-case, four feet high, which stands upon the second part.

The whole is beautifully finished with mahogany, and is highly ornamental, as well as useful, in a variety of ways. It was made

FIG. 2.

by Mr. Dolph, dental furniture manufacturer, of this city, under the supervision of Dr. Smith, at a cost of about $40.00. The Doctor will be pleased to show it to any one who may desire to see it. T.

### ASSOCIATIONS.

The disturbed condition of our country has very much interrupted the regular meetings and business of all professional associations during the last nine months. The attention of the people has been almost wholly absorbed by the magnitude and character of the passing events ; in them our very existence as a nation has been involved.

Many associations of various kinds have, during this time, been postponed to a more convenient season ; others, without any postponement, failed to meet ; others, again, had feeble meetings, and accomplished but little.

The idea that our country, as a whole, is to be plunged into sudden and utter ruin has passed away, and all things have assumed a more steady position. Now associations and societies may meet, with a prospect of accomplishing something.

The organizations of the dental profession have not, perhaps, suffered more from these causes than other bodies. In regard to the meeting of the American Dental Convention, held at New Haven in August last, we are free to acknowledge that we were agreeably mistaken. A good meeting was held, both in regard to the number in attendance and the work done ; and this, too, in the midst of the whirl of excitement that prevailed everywhere. This we hail as one of the best indications of the progress and vigorous growth of our profession.

Other associations have resumed their regular meetings, and gone actively to work ; among which is the Pennsylvania Association of Dental Surgeons, which is meeting monthly, and presenting good reports of discussions, essays, etc.

The New York Dental Association also holds its regular meetings, which are largely attended, and are, doubtless, of great interest.

In association and interchange of views and opinions, the members of the profession in large cities have vastly the advantage over those elsewhere ; and if those thus situated worked together as they should, they would necessarily outstrip in attainments those less fortunately situated ; among the latter, however, we find some of the best students.

Announcements are issued, calling for the meeting of several other dental societies, among which is the Indiana State Dental Association. That call is found in another page of this number of the Register. That meeting, we doubt not, will be a good one, and should be attended by every dentist in the State, and those outside of it, too, as far as possible. There is an accumulation of business, as one regular meeting was postponed.

The Mad River Valley Association holds its regular meeting in January.

The Central Ohio Association, we believe, also holds another meeting soon.

The Mich. Dental Association is announced for the first Tuesday of January.

There are other societies for the meeting of which calls have not been issued ; among which are the Western Dental Society, The Miss. Valley Association, The Northern Ohio Association, and the Kentucky State Dental Association ; all of which, we presume and hope, will hold their regular meetings, and answer the full design of their organization. The springing up of local societies is one of the most favorable indications for the future prosperity of the profession. The tendency of these is to familiarize the members with all practical details ; to establish unity of practice, which is a very important matter ; to do away with prejudice and animosity, and cultivate friendship and good feeling. These associations will ultimately aggregate all in the profession that is truly valuable.

T.

MEETING.

The regular meeting of the Mad River Dental Association will be held at Xenia on the first Thursday of January next, when it is to be hoped there will be a full representation of that part of the country. Some interesting subjects will be presented for discussion, in which all should participate, and by which all would be profited.

T.

A Course of Six Lectures on the Chemical History of a Candle, *after which is added a Lecture on Platinum.* By M. Faraday, F. R. S.—In these lectures is discussed in a very plain, familiar manner, all the elements concerned in the production of the flame of a candle. The chemical properties of each is presented, and the results of these various combinations; it presents this very common though difficult subject in a very understandable and attractive style, one calculated to interest every reader, and lead him into the mazes of chemistry before he is aware of it. There is added a lecture on platinum, the nature of the metal, and the method of working it, which is especially interesting to the metallurgist.

It would be desirable to have the whole range of chemical science brought out in a popular and entertaining way. All will find this volume interesting. For sale by Robert Clarke & Co., of this city.

T.

---

The Physician's Pocket Memorandum for 1862.—By C. H. Cleaveland, M. D.—This little work, arranged for the use of the physician, in addition to its being an engagement and memorandum book, is valuable as a ready reference. It contains many important things in a brief space, and comprehensive manner.

Facts most familiar to the mind will oftentimes, especially during excitement, escape the memory, and something of this kind, to which reference can be made instantly and at all times, will prove a most valuable resource. It contains a list of medicines classified; abbreviations used in prescriptions; a chapter on accidents and emergencies, which should be in the possession of all persons; poisons and antidotes, prescriptions of medicines, post-mortems, etc, This work is certainly one of the best arranged and most convenient for the purpose, with which we are acquainted. T.

---

Dentist's Memorandum for 1861.—This is a book of engagements, and manual of ready reference. It is gotten up with excellent arrangements for engagements; it may also be used as a day book or blotter. The hours for engagements extend from 8 o'clock A. M. to 6 o'clock P. M. It also contains many formulas, which are valuable, and are constantly required for reference. There is also a department for miscellaneous memorandum.

This little work embraces more than anything of the kind we have ever seen, and should be in the possession of every dentist.

It is published, and for sale by Dr. C. H. Cleaveland, of this city, at $1.00. T.

## Figures and Tables

**Fig. 1. f1:**
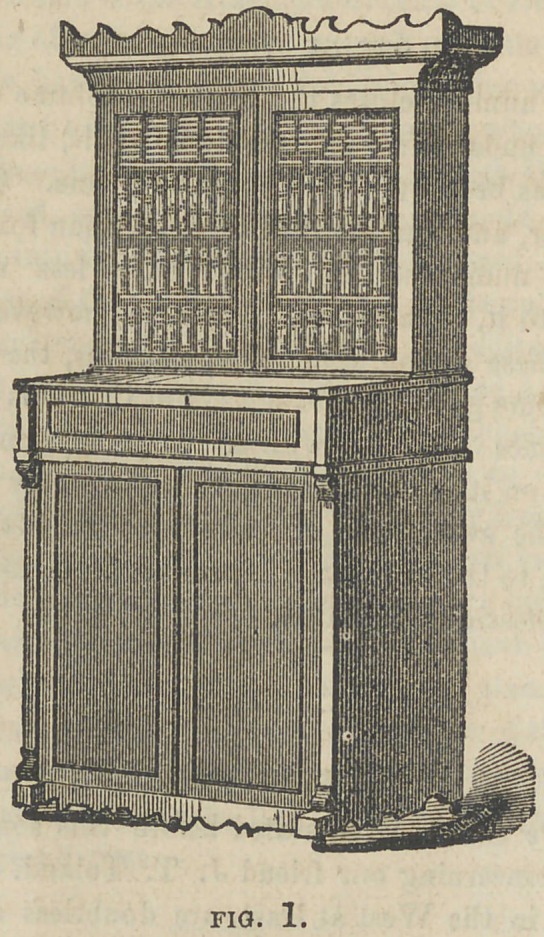


**Fig. 2. f2:**